# Risk Factors of 30-Day All-Cause Mortality in Patients with Carbapenem-Resistant *Klebsiella pneumoniae* Bloodstream Infection

**DOI:** 10.3390/jpm11070616

**Published:** 2021-06-29

**Authors:** Keh-Sen Liu, Yao-Shen Tong, Ming-Tsung Lee, Hung-Yu Lin, Min-Chi Lu

**Affiliations:** 1 Division of Infectious Diseases, Department of Internal Medicine, Show Chwan Memorial Hospital, Changhua 500, Taiwan; liumilka2@gmail.com; 2 Department of Medical Laboratory, Show Chwan Memorial Hospital, Changhua 500, Taiwan; yaushen51@gmail.com; 3 Research Assistant Center, Show Chwan Memorial Hospital, Changhua 500, Taiwan; lee6717kimo@yahoo.com.tw (M.-T.L.); linhungyu700218@gmail.com (H.-Y.L.); 4 Department of Nursing, Hungkuang University, Taichung 433, Taiwan; 5 Division of Infectious Diseases, Department of Internal Medicine, China Medical University Hospital, Taichung 404, Taiwan; 6 Department of Microbiology and Immunology, School of Medicine, China Medical University, Taichung 404, Taiwan

**Keywords:** bacteremia, carbapenemase, carbapenem-resistant *Enterobacteriaceae*, *Klebsiella pneumoniae*, mortality, risk factors

## Abstract

An optimal antimicrobial regimen for the treatment of patients with carbapenem-resistant *Klebsiella pneumoniae* (CRKP) bloodstream infection (BSI) is currently unavailable. This study aimed to identify the appropriate antibiotics and the risk factors of all-cause mortality for CRKP BSI patients. This retrospective cohort study included the hospitalized patients with CRKP BSI. Primary outcome was 30-day all-cause mortality. Cox regression analysis was used to evaluate the risk factors of 30-day mortality. A total of 89 patients were included with a 30-day mortality of 52.1%. A total of 52 (58.4%) patients were treated with appropriate antimicrobial regimens and 58 (65.2%) isolates carried *bla*_KPC-2_ genes. Microbiologic eradication within 7 days (adjusted hazard ratio [HR] = 0.09, *p* < 0.001), platelet count (per 1 × 10^4^/mm^3^, adjusted HR = 0.95, *p* = 0.002), and Pitt bacteremia scores (adjusted HR = 1.40, *p* < 0.001) were independently associated with 30-day all-cause mortality. No effective antimicrobial regimens were identified. In conclusion, risk factors of 30-day mortality in patients with CRKP BSI included microbiologic eradication > 7 days, lower platelet count, and a higher Pitt bacteremia score. These findings render a new insight into the clinical landscape of CRKP BSI.

## 1. Introduction

The rapidly increasing prevalence of antibiotic resistance in *Enterobacteriaceae* is currently a major threat to public health worldwide [1]. Recently, the European Survey of Carbapenemase-Producing *Enterobacteriaceae* (EuSCAPE) Working Group investigated 2703 clinical isolates of carbapeneme-resistant *Enterobacteriaceae* (CRE) submitted from 455 sentinel hospitals in 36 countries and reported that 15% of these were *Escherichia coli* and 85% were *Klebsiella pneumoniae*. Among these Klebsiella isolates, 37% were carbapenemase-producers [2]. Four gene families encoding carbapenemase-production have been identified: *Klebsiella pneumoniae* carbapenemase (KPC), New Delhi metallo-β-lactamase, oxacillinase 48-like, and Verona integron-encoded metallo-β-lactamase [2]. Carbapenem-resistant *Klebsiella pneumoniae* (CRKP) has attracted particular attention since it was first identified as one of the multidrug resistant bacteria strains [3]. Previous studies reported that the KPC-producing *Klebsiella pneumoniae* was independently associated with higher mortality [4,5]. Mortality rate of CRKP bloodstream infection (BSI) is reported to range from 42% to 84% [6]. Mechanistically, the acquisition of genes encoding carbapenemase-production is the predominant mechanism of carbapenem-resistance through inactivation or degradation of carbapenems [7].

Although the innovation of new drugs combating resistant bacteria may create a glimmer of hope, the evolution of drug-resistant genes soon overwhelms progression. For instance, drug resistance-combating ceftazidime-avibactam was first approved under the Generating Antibiotic Incentives Now (GAIN) Act, whereas its clinical use was soon followed by reports of resistance caused by CRKP [8,9,10,11]. Due to the rapid progression and high mortality rate, the role of CRKP can be better understood by an early examination. Nonetheless, the clinical value of CRKP and its related risk factors are yet to be clarified.

Many studies found that CRKP BSI patients treated with combined antibiotics had better survival outcomes than those treated with monotherapy [12,13]. However, potentially effective regimens are varied across studies and those that are available are very limited [14,15,16,17]. Since there is no consensus on optimal antimicrobial regimens for CRKP infection, it is crucial to understand the possible risk factors for unfavourable outcomes and potentially optimal antibiotics when treating CRKP BSI patients. In this retrospective cohort study, we attempted to identify the appropriate antimicrobial regimens and to investigate the risk factors of all-cause mortality for CRKP bloodstream infection (BSI) patients.

## 2. Materials and Methods

### 2.1. Study Design and Patients

This retrospective cohort study was ethically approved by the Institutional Review Board of Show Chwan Memorial Hospital, which is a regional hospital offering an estimated 750 beds in Taiwan (approval number: SCMH_IRB No. 1061202). Hospitalized patients diagnosed with CRKP BSI (number of patients) between June 2014 and August 2017 in Show Chwan Memorial Hospital in Taiwan were included in the study. CRKP BSI was defined by at least one blood-culture positive for a CRKP strain. The patients with polymicrobial BSI and those aged < 20 years were excluded.

### 2.2. Data Collection

All patient data were collected through a retrospective review of medical records. Variables included demographics, medical history, Charlson Comorbidity Index [18], admission history, initial presentations (quick sepsis related organ failure assessment [qSOFA] score [19], systemic inflammatory response syndrome [SIRS] criteria [20], Pitt bacteremia score [21], vital signs, and laboratory data), length of hospitalization, antibiotic regimens, microbiologic results, and drug susceptibilities. Sepsis was defined by qSOFA ≥ 2 [22] and shock was as mean arterial pressure ≤ 65 mmHg [23]. Only antimicrobial agents used for more than 48 h would be included for analysis. Antimicrobial regimens were classified as empiric or definitive. Empiric regimen included the prescription of antibiotics before the blood culture result was available. Definitive regimen referred to prescription of antibiotics based on drug susceptibility results. An appropriate regimen was defined as including one or more in vitro active drugs against the CRKP isolates. Primary outcome was 30-day all-cause mortality, measured from the day on which the first blood culture revealing CRKP was taken. Microbiologic eradication was defined as negative blood cultures for CRKP during follow-up.

### 2.3. Microbiology and Antimicrobial Susceptibilities

The CRKP isolates were collected from the first positive blood culture of each patient. Bacterial identification and antimicrobial susceptibility tests were performed using Phoenix Automated Microbiology System (Becton, Dickinson and Company, USA) and the interpretative criteria of the Clinical and Laboratory Standards Institute (CLSI) guidelines was applied. CRKP was defined as an isolate with a minimum inhibitory concentration (MIC) of ≥2 µg/mL for ertapenem, ≥4 µg/mL for meropenem, or ≥4 µg/mL for imipenem. Due to no available CLSI breakpoints for *Enterobacteriaceae*, MIC breakpoints by the European Committee on Antimicrobial Susceptibility Testing were used for the interpretation of colistin susceptibility (susceptible: ≤2 mg/L, resistant: >2 mg/L) [24] and tigecycline susceptibility was determined by MIC breakpoints by the USA Food and Drug Administration (susceptible: MIC ≤ 2 µg/mL, intermediate: 4 µg/mL, resistant: MIC ≥ 8 µg/mL) [25]. Detection for *bla_KPC_* gene and *bla_OXA-48_* gene were performed by polymerase chain reaction with specific primers, as previously described [26]. Pulse-field gel electrophoresis (PFGE) was used to detect the relatedness of the CRKP strains. The profiles of XbaI macro-restricted fragments of each strain were determined by a standardized PulseNet PFGE protocol [27]. The BioNumerics version 6.6 (Applied Maths, Belgium) was used to analyze the PFGE profiles. The relatedness was based on PFGE profiles using the dice coefficients and the unweighted pair group method with arithmetic mean algorithm. The optimization value and position tolerance were set at 1.5% and 0.75%, respectively.

### 2.4. Statistical Analysis

Continuous variables were presented as median with interquartile range (IQR) and categorical variables were presented as a percentage. The comparison between the survival group and mortality group was performed using the Mann–Whitney U test and Fisher’s exact test for continuous and categorical variables, respectively. Mortality rate was estimated using the Kaplan–Meier method and compared using the log-rank test. A univariate and multivariate Cox proportional hazard model was used to evaluate the risk factors of 30-day mortality. The variables with *p* < 0.1 in the univariate model were manually selected into the multivariate model in a backward stepwise manner. The results of the Cox proportional hazard model were presented as hazard ratio (HR) with 95% confidence interval (CI). A *p*-value of <0.05 was considered statistically significant. IBM SPSS Statistics for Windows, version 24.0 (IBM Corp., Armonk, NY, USA) was used for the statistical analyses.

## 3. Results

### 3.1. Patient Characteristics and Antimicrobial Treatment

As shown in Table 1, a total of 89 patients (59.6% male, median age 75.6 years) with CRKP BSI were included: 43 (48.3%) patients in the 30-day survival group and 46 (51.7%) in the 30-day mortality group. The 7-day, 14-day, and 30-day all-cause mortality of all patients were 32.6%, 43.9%, and 51.7%, respectively. Within 48 h of admission, 23 out of 89 (25.8%) had a positive sign of blood culture testing for CRKP, indicating that it was community acquired. The 66 out of 89 (74.1%) patients who presented a positive sign of blood culture testing for CRKP after 48 h of admission were regarded as nosocomial infections. Survival group patients had a significantly lower Charlson comorbidity index, Pitt bacteremia score, and albumin levels. The proportion of sepsis and septic shock was lower in the survival group, while platelet counts were higher. Pre-existing cardiovascular disease and hospitalizations during the prior year was more uncommon among survivors (Table 1).

The results of antimicrobial susceptibility revealed that 52 (58.4%) were treated with the appropriate antimicrobial regimens, including 9 (10.1%) as empiric therapy and additionally 50 (56.2%) as definite regimens. Colistin (31.5%) and carbapenem (41.6%) were the most commonly prescribed antibiotics. Patients in the survival group had a significant proportion of microbiological eradication within 7 days, as compared to the mortality group (72.1% vs. 13.0%, *p* < 0.001) (Table 2). However, the proportion of appropriate regimen, length of hospitalization, and the pattern of antibiotics utilization did not significantly differ between the two groups (Table 2).

### 3.2. Microbiology Revealed Redcued Proportion of KPC Genes and KPC Cluster II in the Survival Group

**Figure 1:**
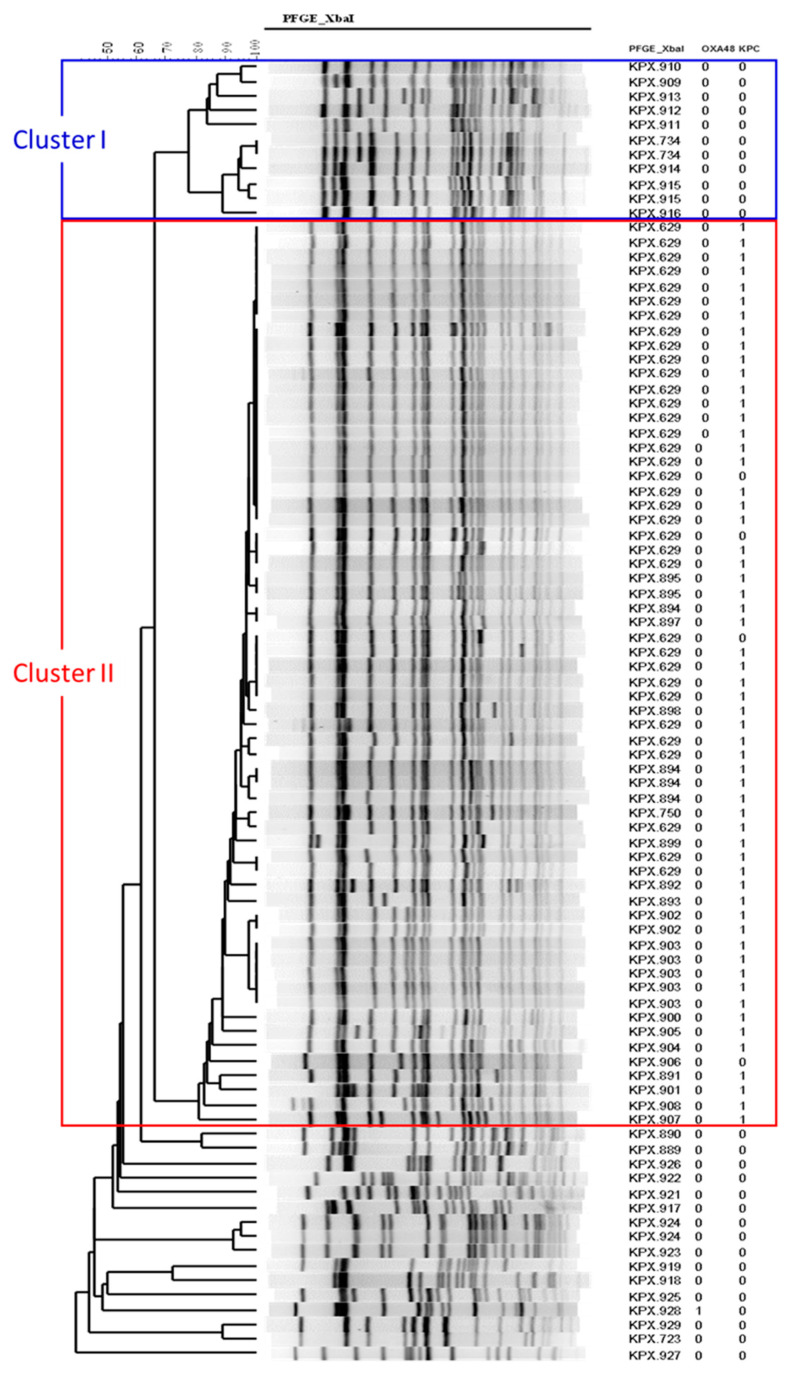
Clonal relatedness of the carbapenemase-producing *Klebsiella pneumoniae* strains. Pulse-field gel electrophoresis (PFGE) was used to determine the profiles of XbaI macro-restricted fragments of each strain were determined by a standardized PulseNet PFGE protocol.

Among the 89 CRKP strains, 58 (65.2%) isolates carried KPC genes, and all were bla_KPC-2_ genes. According to the results of PFGE, two distinct clusters could be identified from the CRKP strains (Figure 1): 11 (12.4%) strains in the cluster I, 62 (69.7%) in cluster II, and others (18.0%). All the strains carrying KPC genes were in cluster II. None of the cluster I and II strains had bla_OXA-48_ genes. Notably, we noted that the survival group had a reduced proportion of KPC genes (51.2% vs. 78.3%, *p* = 0.008) and the cluster II strains (53.5% vs. 84.8%, *p* = 0.006) as compared to the mortality group (Table 1).

### 3.3. Comparison of Antimicrobial Susceptibility

**Figure 2:**
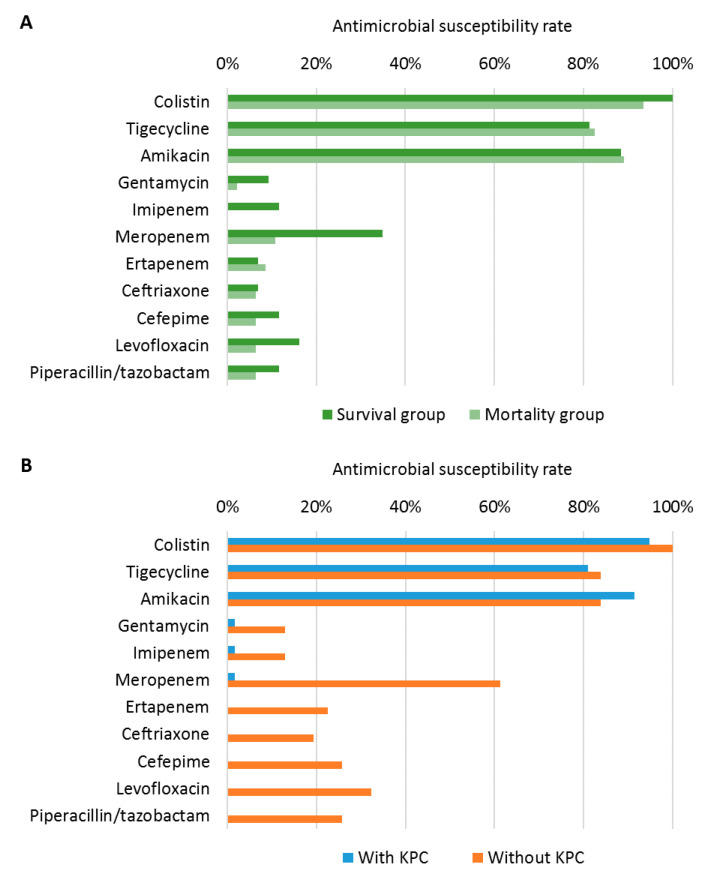
Antimicrobial susceptibility of carbapenemase-producing *Klebsiella pneumonia* isolates according to 30-day mortality (**A**) and the presence of *Klebsiella pneumonia* carbapenemase (KPC) genes (**B**). KPC, *Klebsiella pneumoniae* carbapenemase.

All of the 89 isolates were resistant to at least one of the three carbapenems. For the 89 isolates, colistin exerted the highest susceptibility rate (96.6%), followed by amikacin (88.8%), tigecycline (82.0%), meropenem (22.5%), levofloxacin (11.2%), ertapenem (7.9%), and imipenem (5.6%) (Figure 2A). The pattern of drug susceptibility in the survival group differed from that of the mortality group (Figure 2A). The pattern difference was observed between CRKP strains with and without KPC genes (Figure 2B). The survival group had a significantly higher susceptibility rate of imipenem (11.6% vs. 0%, *p* = 0.023) and meropenem (34.9% vs. 10.9%, *p* = 0.01) than the mortality group (Figure 2A). Except for colistin, tigecycline, and amikacin, the susceptibility rate of all drugs were significantly lower in the CRKP strains with KPC genes than in the strains without KPC genes.

### 3.4. Risk Factors of 30-Day All-Cause Mortality

**Figure 3:**
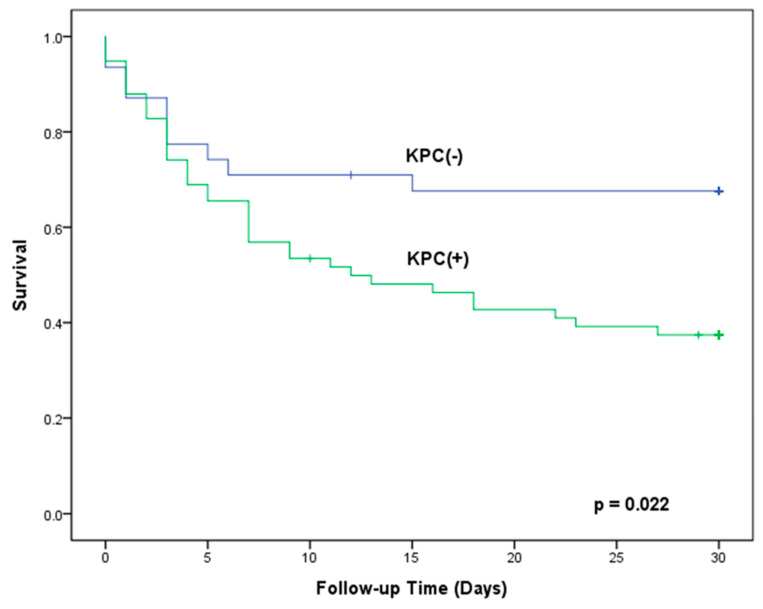
Kaplan-Meier curve showing the 30-day in-hospital survival for bacteremic patients with carbapenem-resistant *Klebsiella pneumoniae* (CRKP) carrying KPC gene as compared to CRKP not carrying KPC gene.

For patients with CRKP BSI, multivariate analysis revealed that microbiologic eradication within 7 days (adjusted HR = 0.09, *p* < 0.001), platelet count (per 1 × 10^4^/mm^3^, adjusted HR = 0.95, *p* = 0.002), and Pitt bacteremia score (adjusted HR = 1.40, *p* < 0.001) were independently associated with 30-day all-cause mortality. The univariate Kaplan-Meier method revealed that CRKP patients with KPC genes had significantly higher 30-day mortality than those without KPC genes (62.6% vs. 32.4%, *p* = 0.022) (Figure 3). However, the predictive role of the presence of KPC genes was not confirmed by the multivariate model. None of the antimicrobial regimens was significantly associated with 30-day mortality (Table 3).

## 4. Discussion

CRKP BSI is a clinical challenge as no effective antimicrobial regimens are currently available. This study observed a 30-day all-cause mortality of 52.1% in patients with CRKP BSI. The lack of microbiologic eradication within 7 days, a lower platelet count, and a higher Pitt bacteremia score were independently associated with higher 30-day mortality. Notably, the predictive role of KPC genes and appropriate antibiotic regimens were not identified in this study.

All the KPC genes detected in this study were *bla_KPC-2_*, which was consistent with the previous nationwide surveillance in Taiwan that *bla_KPC-2_* accounted for the majority of KPC genes [28]. However, in the present study, the prevalence of *bla_KPC-2_* gene among CRKP strains was 65.2%, much higher than the previous prevalence of 36.2% in 2011–2015 [29]. The results suggested the KPC-2-producing CRKP strains disseminate rapidly at an alarming rate in Taiwan. In addition, this study reveals that non-KPC-producing strains present high susceptibility to imipenem, meropenem, ertapenem, ceftriaxone, cefepime, levofloxacin, and piperacillin/tazobactam, as compared to that of KPC-producing strains. Although the results might suggest the role of KPC in the treatment of CRKP BSI, multivariate analysis did not find an association of KPC with mortality. Wang et al. reported similar results that KPC-gene positive and negative strains had different MICs of antibiotics, but mortality did not differ between these two CRKP strains [30]. Some studies suggested CRKP infection as an important risk factor of hospital mortality [4,5]; however, the role of KPC deserves further investigations for patients with CRKP BSI.

This study revealed that a higher Pitt bacteremia score was independently associated with 30-day mortality in patients with CRKP BSI. The result was comparable with the study of Shen et al. and Xiao et al. [31,32] Gomez-Simmonds et al. and Lee et al. also reported that the patients with Pitt bacteremia score > 4 had significantly higher mortality [15,17] Additionally, our study revealed an association of lower platelet count with higher mortality, which might also reflect the severity of illness [33]. Some studies used different tools to evaluate the severity of illness at the presence of CRKP BSI, for example, APACHE II score; a higher APACHE II score was reported to be independently associated with 30-day mortality [13,34]. All these results supported the predictive value of the severity of illness for 30-day morality in patients with CRKP BSI.

Both our results and that of Nguyen et al. revealed that the patients with microbiologic eradication within 7 days had a significantly better survival rate [35]. Falcone et al. further evaluated the effect of time to appropriate antibiotic therapy on 30-day mortality and showed that time to appropriate antibiotic therapy was an independent predictor of 30-day mortality in patients with CRKP BSI. Falcone et al. suggested that appropriate antibiotic therapy was preferably initiated within the first 24 h after collection of the blood culture [36]. From our study and the previous research, the importance of timely control of the disease is evident.

Many studies have attempted to identify the best combination of antibiotics for the treatment of CRKP BSI, but the results are mixed and there is no consistent conclusion [14,15,16,17]. Our results revealed that the most susceptible drug was colistin, followed by tigecycline and amikacin [13,15,31,35,37]. Medeiros et al. observed that the combination of colistin and amikacin could provide survival benefits for the patients with CRKP BSI [38]. However, both colistin [39] and amikacin [40] are nephrotoxic agents. Most patients with CRKP BSI are older, have multiple comorbid diseases, and frequently present with septic shock [13,35,41]. The use of colistin or amikacin, or in combination, can further aggravate nephrotoxicity [39,40]. This combination is reasonable on the basis of only antimicrobial susceptibility without consideration of patients’ condition and drug adverse effects. Clinicians are usually reluctant to use the combination because of the high risk of renal injury, which may lead to dialysis and increase subsequent morbidity and mortality. Although tigecycline causes minimal organ toxicity, the use of tigecycline for Gram-negative bacteremia remains controversial. The serum concentrations provided by standard doses of tigecycline are below the MICs of most Gram-negative pathogens [42]. Therefore, the most common combinations are colistin/tigecycline, aminoglycoside/tigecycline, colistin/carbapenem, aminoglycoside/carbapenem, and a combination of three drugs (e.g., colistin/tigecycline/carbapenem) [12,13]. However, it lacks consensus on the best combination for the treatment of CRKP BSI. Ceftazidime-avibactam is a new combination of third generation cephalosporin and non-β-lactam β-lactamase inhibitor, and has promising in vitro activity against many Gram-negative pathogens, including KPC-producing *Enterobacteriaceae* [43]. It is hoped that the new drugs will not be misused or overused to avoid the development of antibacterial resistance [44].

The major caveat of this study is the observational design, in which potential reporting bias and selection could not be avoided. Secondly, all patients were diagnosed and treated in a single institute, which limited the external validity of the results. Thirdly, this study focused on the patients with CRKP BSI instead of other types of infection, such as urinary tract infection, pneumonia, or intra-abdominal infection. Fourthly, the definition of appropriate regimens in this study was the inclusion of one or more efficient antibiotics, instead of two or more, and the time to appropriate regimens was not evaluated. This might explain the results that the use of appropriate regimen was not significantly associated with 30-day mortality.

## 5. Conclusions

This retrospective cohort study observed that the risk factors of 30-day all-cause mortality in patients with CRKP BSI included microbiologic eradication > 7 days, a lower platelet count, and a higher Pitt bacteremia score. These findings render new insights into the clinical landscape of CRKP BSI.

## Figures and Tables

**Table 1:** Demographics and baseline characteristics in patients with carbapenem-resistant *Klebsiella pneumoniae* bloodstream infection according to 30-day mortality.

Variables	All (*n* = 89)	Survival Group (*n* = 43) (48.3%)	Mortality Group (*n* = 46) (51.7%)	*p* Value
Demographics				
Male; n (%)	53 (59.6%)	26 (60.5%)	27 (58.7%)	1.000
Age; y/o (IQR)	75.6 (63.8–83.7)	74.5 (62.3–83.4)	80.5 (65.8–85.2)	0.153
BMI; kg/m^2^ (IQR)	21.3 (18.3–25.9)	20.8 (18.2–24.8)	21.4 (19.2–30.0)	0.223
Comorbidities				
Charlson comorbidity index; score (IQR)	8 (6–10)	7 (6–9)	8 (7–11)	0.008
Diabetes mellitus; n (%)	53 (59.6%)	24 (55.8%)	29 (63.0%)	0.523
Cardiovascular disease; n (%)	57 (64.0%)	34 (79.1%)	23 (50.0%)	0.008
Chronic obstructive pulmonary disease; n (%)	21 (23.6%)	9 (21.4%)	12 (26.1%)	0.628
Chronic liver disease; n (%)	15 (16.9%)	4 (9.5%)	11 (23.9%)	0.092
Chronic kidney disease; n (%)	15 (16.9%)	9 (21.4%)	6 (13.0%)	0.397
Malignancy; n (%)	18 (20.2%)	7 (16.7%)	11 (23.9%)	0.439
Steroid use ≥ 3 months; n (%)	15 (16.9%)	4 (9.5%)	11 (23.9%)	0.092
Immunocompromised condition; n (%)	8 (9.0%)	3 (7.1%)	5 (10.9%)	0.716
Events in the prior year				
Hospitalization; events (%)	65 (73.0%)	36 (85.7%)	29 (64.4%)	0.028
Admitted to intensive care units; events (%)	30 (33.7%)	16 (38.1%)	14 (30.4%)	0.504
Nursing home residence; events (%)	31 (34.8%)	14 (32.6%)	17 (37.0%)	0.824
CRKP colonization; events (%)	14 (15.7%)	7 (16.7%)	7 (15.2%)	1.000
Surgery; events (%)	24 (27.0%)	12 (27.9%)	12 (26.1%)	1.000
Initial presentation				
Pitt bacteremia score; score (IQR)	4 (2–6)	3 (1–4)	6 (4–8)	< 0.001
SIRS; score (IQR)	3 (2–4)	3 (2–3)	3 (2–4)	0.273
SIRS ≥ 2; n (%)	76 (85.4%)	35 (81.4%)	41 (89.1%)	0.375
qSOFA; score (IQR)	2 (1–3)	1 (1–2)	2 (2–3)	< 0.001
Sepsis; n (%)	54 (60.7%)	18 (41.9%)	36 (78.3%)	0.001
Septic shock; n (%)	27 (30.3%)	7 (16.3%)	20 (43.5%)	0.006
Body temperature ≥38 °C; n (%)	54 (60.7%)	27 (62.8%)	27 (58.7%)	0.828
White blood cell count (10^3^/mm^3^); count (IQR)	13.2 (8.7–17.8)	13.7 (10.1–17.8)	10.7 (6.4–18.0)	0.249
Hemoglobin (g/dL); value (IQR)	9.8 (9.0–10.6)	9.8 (9.1–11)	9.7 (8.6–10.5)	0.352
Platelet count (10^4^/mm^3^); count (IQR)	12.4 (5.2–23.8)	17.5 (10.8–26.3)	8.15 (3.8–18.2)	0.002
Creatinine (mg/dL); value (IQR)	1.3 (0.8–2.8)	1 (0.7–2.8)	1.4 (0.9–2.8)	0.088
Albumin (g/dL); value (IQR)	2.6 (2.2–2.9)	2.9 (2.5–3.0)	2.4 (2.1–2.7)	< 0.001
C-reactive protein (mg/dL); value (IQR)	11.6 (4.9–19.4)	10.8 (3.6–19.3)	12.7 (8.0–19.6)	0.256
Microbiology				
Presence of KPC gene; n (%)	58 (65.2%)	22 (51.2%)	36 (78.3%)	0.008
Clonal relatedness of CRKP strain				0.006
Cluster I; n (%)	11 (12.4%)	8 (18.6%)	3 (6.5%)	
Cluster II; n (%)	62 (69.7%)	23 (53.5%)	39 (84.8%)	
Others; n (%)	16 (18.0%)	12 (27.9%)	4 (8.7%)	

Abbreviations: BMI, body mass index; CRKP, carbapenem-resistant *Klebsiella pneumoniae*; IQR, interquartile range; KPC, *Klebsiella pneumoniae* carbapenemase; qSOFA, quick sepsis related organ failure assessment; SIRS, systemic inflammatory response syndrome.

**Table 2:** Treatment in patients with carbapenem-resistant *Klebsiella pneumoniae* bloodstream infection according to 30-day mortality.

Variables	All (*n* = 89)	Survival Group (*n* = 43)	Mortality Group (*n* = 46)	*p* Value
Appropriate antimicrobial regimen; n (%)	52 (58.4%)	28 (65.1%)	24 (52.2%)	0.283
Colistin included; n (%)	28 (31.5%)	10 (23.3%)	18 (39.1%)	0.006
Amikacin included; n (%)	13 (14.6%)	9 (20.9%)	4 (8.7%)	0.336
Carbapenem included; n (%)	37 (41.6%)	17 (39.5%)	20 (43.5%)	0.124
Tigecycline included; n (%)	3 (3.4%)	0 (0%)	3 (6.5%)	0.092
Monotherapy; n (%)	12 (13.5%)	11 (25.6%)	1 (2.2%)	0.003
Appropriate empiric regimen; n (%)	9 (10.1%)	3 (7.0%)	6 (13.0%)	0.487
Appropriate definitive regimen; n (%)	50 (56.2%)	28 (65.1%)	22 (47.8%)	0.135
Microbiologic eradication within 7 days; n (%)	37 (41.6%)	31 (72.1%)	6 (13.0%)	<0.001
Length of hospitalization; days (IQR)	21.5 (13.0–34.0)	21.5 (14.0–40.0)	21.5 (13.0–33.0)	0.483

Abbreviations: CRKP, carbapenem-resistant *Klebsiella pneumoniae*; IQR, interquartile range; KPC, *Klebsiella pneumoniae* carbapenemase.

**Table 3:** Multivariate analysis for risk factors of 30-day mortality in patients with carbapenem-resistant *Klebsiella pneumoniae* bloodstream infection.

Variables	Adjusted Hazard Ratio	95% Confidence Interval	*p*
Microbiologic eradication within 7 days (yes vs. no)	0.09	0.03–0.26	<0.001
Platelet count (per 1 × 10^4^/mm^3^)	0.95	0.92–0.98	0.002
Pitt bacteremia score (per 1 unit)	1.40	1.21–1.61	<0.001

## Data Availability

The data used to support the findings of this study are available from the corresponding author upon request.
